# A Rare Case of an Occult Primary Tumor With a Profile of Colon Cancer and Synchronous Metastasis in the Lung, Liver, Bone, and Cerebellum: A Case Report and Literature Review

**DOI:** 10.7759/cureus.47824

**Published:** 2023-10-27

**Authors:** Ramona Abrudan, Luca Abrudan, Ovidiu Pop, Dana Carmen Zaha

**Affiliations:** 1 Oncology, Faculty of Medicine and Pharmacy, Doctoral School of Biomedical Sciences, University of Oradea, Oradea, ROU; 2 Radiation Oncology, Radiotherapy Laboratory, County Clinical Emergency Hospital Bihor, Oradea, ROU; 3 Pathology, Department of Morphological Sciences, Faculty of Medicine and Pharmacy, University of Oradea, Oradea, ROU; 4 Laboratory Medicine, Department of Preclinical Disciplines, Faculty of Medicine and Pharmacy, University of Oradea, Oradea, ROU

**Keywords:** osteolytic bone lesion, cerebellum, metastasis, colorectal cancer, cancer of unknown primary site

## Abstract

Occult primary tumors, or cancers of unknown primary site (CUP), are an oncological pathology characterized by the presence of metastases but without being able to determine the presence of the primary tumor. These types of tumors are very rare, and they pose challenges for diagnosis and treatment. Colorectal cancer is the most common type of malignant tumor worldwide and the second most common cause of death. The most common sites of metastasis in colorectal cancer are hepatic and pulmonary. Relatively rare, patients develop brain and bone metastasis. We reported a rare case of an occult primary tumor with a profile of colon cancer and synchronous metastasis in the lung, liver, bone, and cerebellum developed in a woman who was only 51 years old.

## Introduction

Cancers of unknown primary site (CUP) or occult primary tumors are metastatic cancers, histologically confirmed with no identified primary site following standard pretreatment evaluation [1,2]. This type of tumor is rare and accounts for about 3-5% of all cancers diagnosed worldwide [2,3].

The initial evaluation procedure includes a detailed history and physical examination, a blood draw with basic blood and biochemical analyses, either a CT scan of the chest, abdominal, and pelvis regions with intravenous contrast substances or MRI scans of the head, neck, chest, abdomen, and pelvis with an intravenous contrast agent, and mammography in females. All these imaging methods should be used according to the symptoms and findings of the patient. [1,2,4]. Further tests are indicated based on clinical and pathological findings, and these are tumor markers, gastroscopy, colonoscopy + biopsy, bronchoscopy + biopsy, histopathological + immunohistochemistry examinations, and whole body F2-fluoro-2-deoxy-D-glucose (FDG) - positron emission tomography (PET) - CT examination [2]. These investigations led to better detection of primary tumors, which is quantified by the declining incidence of CUP from between 3% and 5% to 1% and 2% of all tumor diagnoses [5]. Most patients with CUP present multiple metastatic sites, including the lung, liver, lymph nodes, abdominal cavity, bone, and brain, with a specific pattern of early dissemination and aggressiveness [1,2]. Median survival is between 8 and 12 months [1].

Colorectal cancer (CRC) is the third most common type of malignant tumor worldwide; in 2020, almost 2 million patients were diagnosed, and it is also the second most common cause of death, with almost 1 million deaths per year [6,7] The incidence of CRC in a sex-dependent manner revealed that in men, it is the third most common type of cancer after pulmonary and prostate cancer, and in women, it is the second most common type of cancer after breast cancer. The incidence of CRC in an age-dependent manner is between 68 and 72 years old [6]. The last statistics showed that the incidence in older patients decreased by almost 3.6%, but the incidence is increasing alarmingly, with almost 11% in patients under 50 years old [6]. It showed that almost 25% of patients present at the diagnosis had distant metastasis, and the other 25% would suffer from distant metastasis [8]. The most common sites of metastasis in colorectal cancer are hepatic, pulmonary, and relatively rare. Patients develop brain metastasis in about 0.6-3.2% of cases [9] and bone metastasis in about 10-15% of cases [10]. The prognosis of patients with colon cancer and bone metastasis is poor, with a five-year survival rate of ˂5% [10]. The prognosis for patients with CRC and brain metastasis is poor too, with a 2.6 to 7.4 month median survival rate, and only a few patients survive more than one year [10]. It was demonstrated in the literature that the most affected bones from metastasis in a case of colorectal cancer are the spine, pelvis, and long bones [10].

We aimed to report a case of an occult primary tumor with synchronous cerebellar, bone, lung, and liver metastasis.

## Case presentation

A 51-year-old female patient with a history of cholecystectomy (performed as a result of acute cholecystitis due to gallstones) presented to the Emergency Medicine Hospital for symptoms of intracranial hypertension: diffuse headaches, nausea, and vomiting appeared in the last week before the presentation.

Laboratory examinations revealed a normal complete blood count and liver and kidney function, but the serum levels of tumor markers CEA: 176.92 U/L (NV: 0-5 U/L) and CA19-9: 1025.89 U/L (NV: 0-37 U/L) were significantly increased.

**Figure 1:**
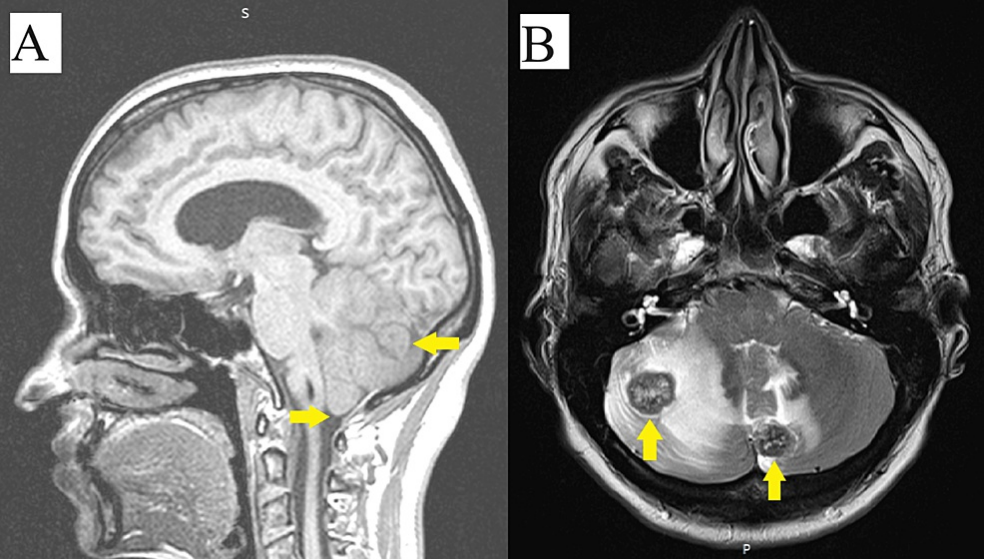
MRI scan. (A) the right yellow arrow showed cerebellar metastasis and the left yellow arrow a slight herniation of cerebral tonsils through the foramen magnum – sagittal section T1 and (B) two cerebellar metastases with perilesional edema – transverse section T2.

A brain MRI (spatial resolution: 384 x 384 and slice thickness: 4/0/4mm) with intravenous contrast showed six well-defined masses, four on the right cerebellum hemisphere with a maximum diameter of 2.1 cm × 1.8 cm, and in the left cerebellum hemisphere, two well-defined masses with a maximum diameter of 1.6 cm × 1.8 cm (Figure 1B). These lesions are associated with moderate perilesional edema, a slight compression on the fourth ventricle, a slight herniation of the cerebral tonsils through the foramen magnum by 3-4 mm, and triventricular hydrocephalus (Figure 1A).

**Figure 2:**
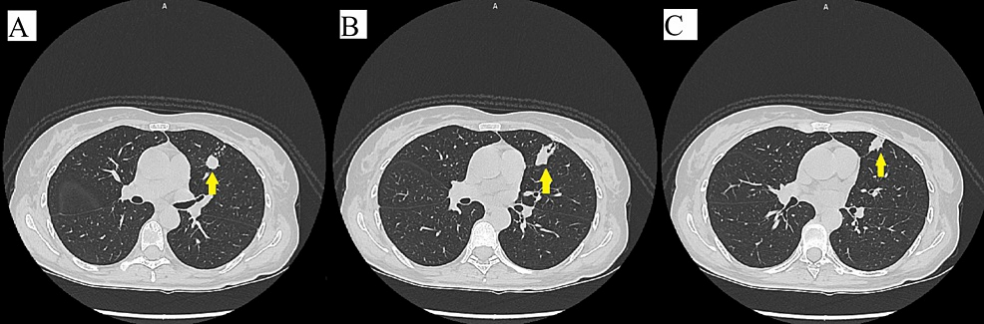
Chest CT-scan (spatial resolution: 512 × 512 and slice thickness: 1.25 mm). (A) Inhomogeneous lung opacity in the left superior lobe, (B) lung opacity with irregular edges, and (C) lung opacity with spiculiform extensions to the pleura CT scan with intravenous contrast substance – axial images.

**Figure 3:**
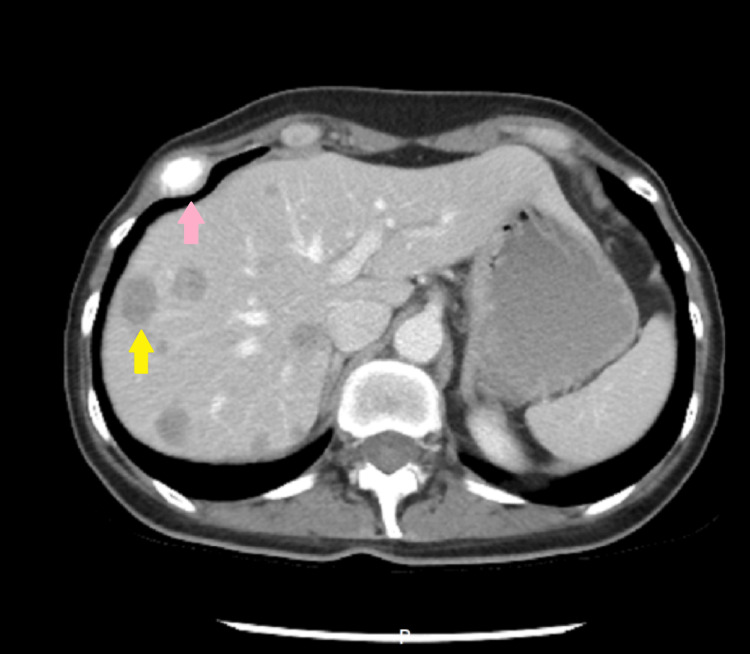
Abdominal CT-scan (spatial resolution: 512 × 512 and slice thickness: 5 mm). One of the multiple liver metastases with a yellow arrow and with pink arrow an osteolytic lesion on the seventh rib CT scan with intravenous contrast substance – axial image.

**Figure 4:**
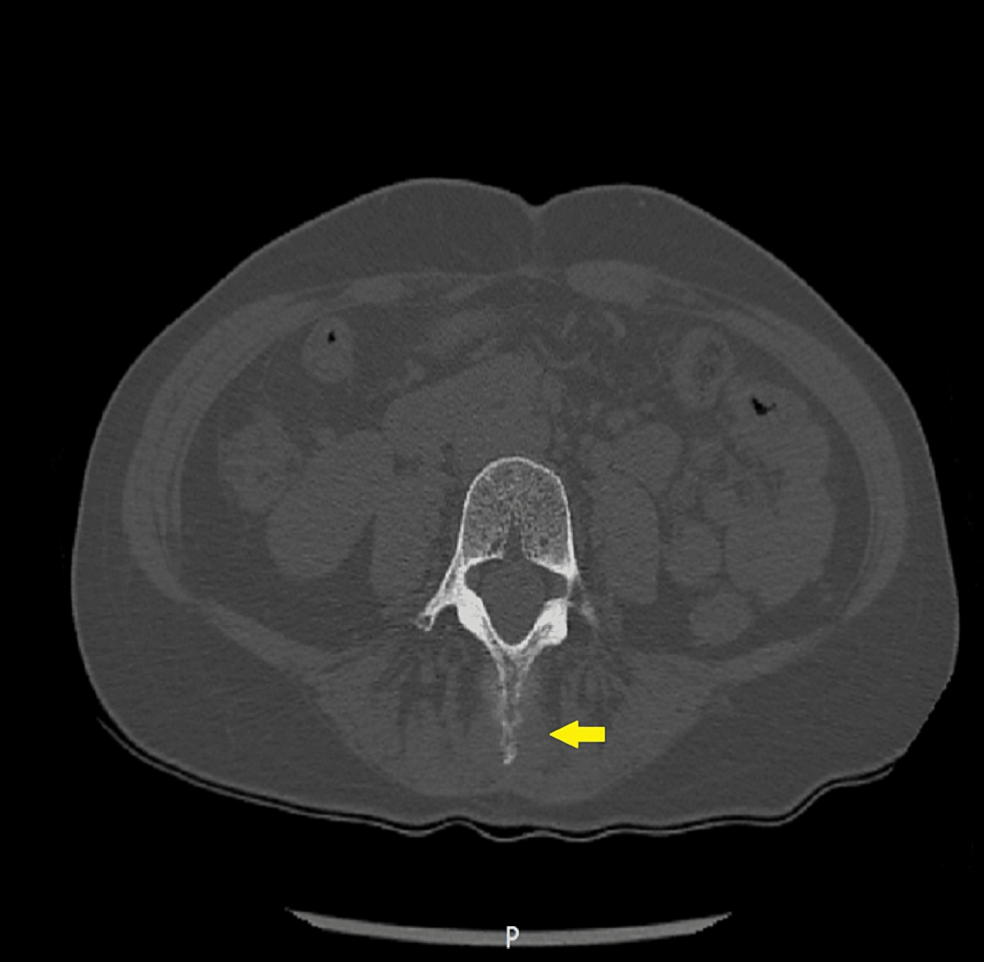
Abdominal CT-scan (spatial resolution: 512 × 512 and slice thickness: 1.25 mm). An osteolytic lesion on spinous process of lumbar fourth vertebra with soft tissue expansion CT scan with intravenous contrast substance – axial image.

Due to multiple cerebellar metastases associated with herniation in the foramen magnum, surgical intervention or even a biopsy was out of the question because, after the neurosurgical consultation, the patient refused the surgical intervention. Then it was decided to investigate the whole body in detail in order to determine the origin of the primary tumor. Before any investigation, an anti-edema treatment was administered: dexamethasone 8 mg/2 ml: 2 times/day; mannitol 200 mg/ml: 1 time/day; levetiracetam 500 mg: 2 times/day. A CT scan with intravenous contrast of the chest, abdominal, and pelvis revealed inhomogeneous lung opacity in the left superior lobe with probable areas of necrosis inside, irregular edges, and spiculiform extensions to the pleura with an axial diameter of approximately 2.15/1.5 cm (without spiculiform extensions) and cranio-caudal diameter of 1.8 cm (Figure 2A-2C). Liver with multiple well-defined masses with a diameter of 2.2 cm (Figure 3). Osteolytic lesions on the right ribs R1, R7 (Figure 3), vertebral thoracic and lumbar bodies, and L4 are soft tissue expansion adjacent to the spinous process (Figure 4), right sacrum bone, and posterior left iliac bone.

**Figure 5:**
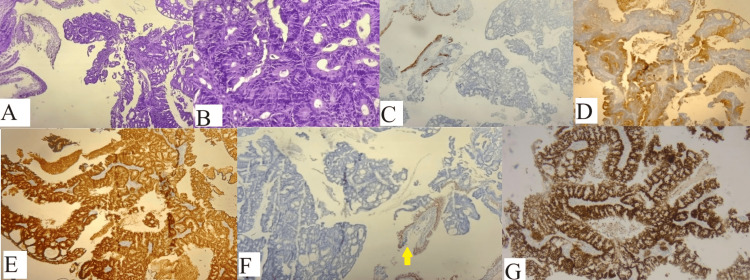
(A) Lung trans-thoracic needle-biopsy showing normal bronchia in the left side with atypical glandular proliferation to the right (HE 5×); (B) atypical glandular proliferation (HE 20×); (C) positive expression for CK7 for the normal respiratory type bronchial mucosa to the left and negative for the proliferation at the right side of the picture; (D) positive expression for CK20 for the atypical proliferation; (E) positive expression for CDX2; (F) negative expression for TTF1 but with positive control at level of the respiratory type bronchial mucosa (yellow arrow); (G) positive expression for SATB2.

According to the CT evaluation, it was decided to perform a bronchoscopy, which revealed trachea in the third distal part of the left lateral wall, an extrinsic compression along three cartilaginous rings, and in the left superior lobe, a voluminous endoluminal, pedicle, and floating proliferative mass that obstructs almost completely the superior lobar bronchus (biopsy). Pathological evaluation was reported as a proliferation of atypically glandular structures (Figure 5A-5B). The immunohistochemical analysis was interpreted as follows: CK7 (Figure 5C) and TTF1 (Figure 5F) were negative for atypical glandular proliferation and positive for CK20 (Figure 5D), CDX2 (Figure 5E), and SATB2 (Figure 5G). All aspects described so far incline toward a secondary determination in the lungs of an intestinal-type adenocarcinoma, moderately differentiated, with an enteric origin. Also, the genetical analysis for the KRAS mutation was performed: an activator mutation in exon 2 c.35G˃T (p.(Gly12Val)).

Considering that the histopathological and immunohistochemical profile of the lung biopsy reveals a cancer of gastrointestinal origin, a flexible colonoscopy is required, which detects 30 cm from the anal verge two diametrically opposite lesions, one elevated and measuring almost 15 mm with central ulceration and the other measuring almost 10 mm with central ulceration (biopsy from both lesions). The histopathological examination of the colon biopsy showed superficial fragments of intestinal mucosa presented in the lamina propria, moderate and diffuse lymphocytes, plasma cells, and a few dysplastic glandular structures.

Furthermore, for the bone lesions, whole-body scintigraphy with 99 mTc MDP-500 mbq showed hyperfixation areas in the left parietal bone, multiple lesions on the vertebral bodies, the right ribs of C1 and C7, the right sacrum bone, the right iliac bone, the right acetabulum, the right ischium bone, the proximal third of the right humerus bone, and the left femoral bone.

**Figure 6:**
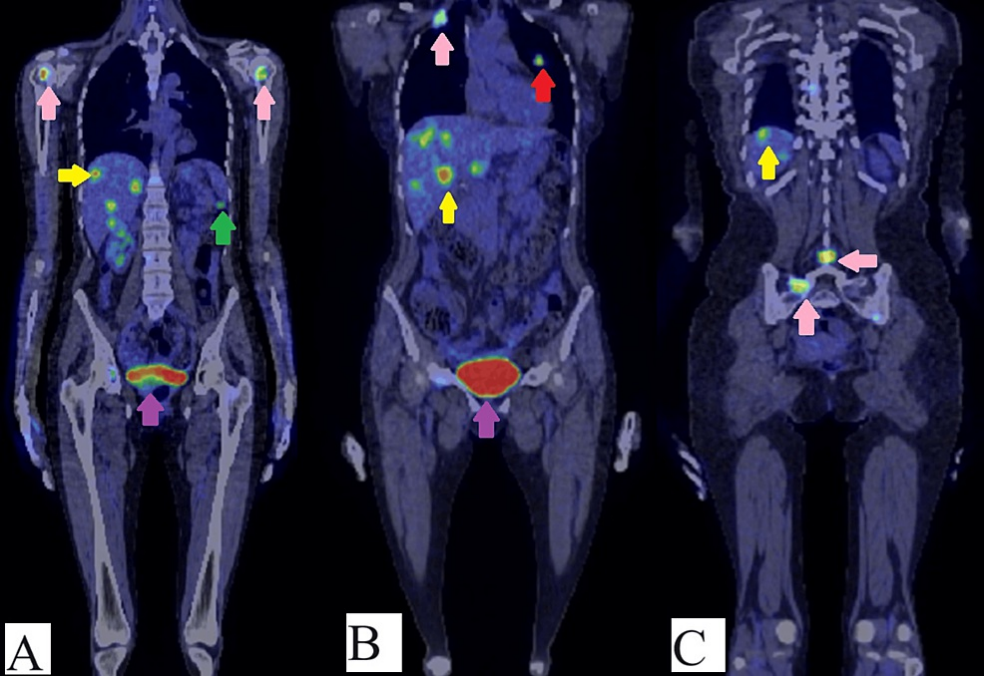
PET-CT images. (A) Multiple osteolytic lesions with an abnormal increase in metabolism on both heads of the humerus (pink arrow), in the left colic flexure an abnormal increase in metabolism (green arrow), abnormal increase metabolism in the liver - multiple metastases (yellow arrow) and urinary bladder, FDG accumulation (purple arrow); (B) abnormal increase metabolism: in the lung (red arrow), in liver (multiple metastases) (yellow arrow), first rib (osteolytic lesion) (pink arrow) and urinary bladder, FDG accumulation (purple arrow); (C) abnormal increase metabolism on pelvic bones and L4 spinous vertebra, process extended in the soft adjacent tissue (pink arrows) and liver metastases (yellow arrow).

According to the pathological findings, a PET-CT scan was performed with an FDG (fluorodeoxyglucose) intravenous contrast agent, and the results revealed a hypodense mass of 35/20 mm with a deficit of substance in the right cerebellum hemisphere; a lung mass that measures 20/11mm in the left superior lobe, with spiculiform edges to the pleura (Figure 6B); liver with multiple well-defined masses localized in both lobes of the liver ranging in size between 8 and 30 mm (Figure 6A-6C); in the left colic flexure presented a mass of 15 mm with an abnormal increase in metabolism (Figure 6A); multiple osteolytic lesions with an abnormal increase metabolism on the spine, pelvic bones, both heads of the humerus, 1 and 6 rib on the right side. The lesions form the right 1 rib and vertebral L4 (Figure 6C) spinous process extended in the soft adjacent tissue.

With all these data, it was initiated as the first-line treatment regimen: Bevacizumab 7.5 mg/kg i.v. day 1 + CAPEOX (oxaliplatin 130 mg/m^2^ i.v. day 1 + capecitabine 1000 mg/m^2^ twice daily p.o. for 14 days) + zoledronic acid and also external beam radiation therapy (EBRT): whole brain: total dose 30 Gy/10 fractions, energy: 15 mV.

## Discussion

In a meticulous search in the literature (PubMed, Web of Science), we did not find any similar cases to the one detailed here; therefore, we have tried to correlate some particularities of the case with the latest data in the literature, on the one hand, the aspects related to the occult primary tumor and, on the other hand, the brain metastases, especially the one related to the cerebellar and bone metastases in colorectal cancer.

The main important risk factors for occult primary tumors are smoking, type 2 diabetes, autoimmune diseases, family predisposition, high body mass index, low socio-economic status, and black ethnicity [2]. Compared to these data, our patient does not present any of the risk factors mentioned above.

In a study led by Yang et al., they enrolled 32 patients with tumors of unknown primary site, and they found that the most common symptoms in patients with primary occult tumors are: anemia, fever, edema/multiple serous cavity effusion, abdominal distension, or enlargement of the lymph node [3]. In our case, the patient did not show any of these symptoms; she presented symptoms due to brain metastasis, more precisely symptoms of intracranial hypertension: diffuse headache, nausea, and vomiting.

The median age at diagnosis of occult tumors is between 60 and 75 years old and is slightly higher in men than in women [1-3], but in the study by Yang et al., the median age was 52 years old [3]. Thus, compared with these findings, our patient is much younger according to the literature data, but compared with the study by Yang et al., the patient's age is similar. Regarding gender, in all the studies, a slightly higher frequency of males was reported, but our patient is a female.

In terms of tumor histology, the most common types are well- and moderately differentiated adenocarcinomas in almost 60% of cases, poorly differentiated or undifferentiated adenocarcinomas in 30% of cases, squamous cell carcinoma in 5%, and undifferentiated malignant tumors [11]. According to the data examined from the literature, the histopathological result obtained in our hospital coincides with an adenocarcinoma.

In the literature, few studies have focused on brain and bone metastasis in colorectal cancer, referred to as metachronous metastasis, a normal event that occurred during the course of CRC, and very rare cases reported as synchronous metastasis [8,10,12,13]. Our search of the literature showed that the median age at the diagnosis of brain metastasis in colorectal cancer was between 56 and 73 years old [8]. Another observation was that more men develop brain metastasis than women [8]. In patients diagnosed with CRC and bone metastasis, it has been reported that the median age was 64 years old, and the male gender was more frequently affected than the female gender [10]. Based on these statistics, our patient is younger, and her gender is female. From the perspective of metastasis, our patient presented with synchronous metastasis in the lung, liver, bones, and cerebellum.

Kawamura et al. and Santini et al. reported in both studies that the most specific sites of bone metastasis from CRC were the spine, followed by the pelvis, long bones, and other sites such as the hand, feet, and skull [10,14]. However, in the study by Zhenghong et al., the most common site of bone metastasis in CRC patients was the pelvis, followed by the lumbar region, thoracic vertebra, sacral vertebra, and rib [15]. Compared with the aforementioned data, our case presents the most frequent sites of bone metastasis, including the spine, pelvic bones, both heads of the humerus, left femoral bone, left parietal bone, and also atypical in two ribs. Moreover, in the studies by Santini et al. and Zhenghong et al., the most common types of metastatic bone lesions were osteolytic, followed by mixed and osteoblastic lesions [14]. Similar to the previously mentioned study, in our case, all bone lesions are osteolytic.

Another aspect noted in the literature was that the Ras mutation, especially the KRAS mutation, in CRC was significantly more common in lung and brain metastasis [16]. Similar to the literature, our patient presented a KRAS mutation in exon 2, and the metastases were also in the lung and brain.

The scientific literature showed that, in general, studies have focused on brain metastasis in colorectal cancer, but we found only one study that focused only on cerebellar metastases, but in different types of cancers [9]. According to the study by Yoshida et al., the most common regions of primary tumors with cerebellar metastases are the lung, breast, gastrointestinal tract, and others, and regarding the histopathological examination, the most common types are adenocarcinomas, followed by squamous cell carcinoma and unclassified carcinoma [9]. Moreover, another important aspect to mention refers to the multiple cerebellar metastases that were present in more patients than the single ones [9]. Similar to the exposed data, the patient presents several cerebellar metastases and a histopathological profile of adenocarcinoma, but in contrast, the origin of the cerebellar metastases comes from an occult tumor with a profile of colon cancer. We found in the literature a case of colon cancer with liver metastases and one cerebellar metastasis [17]. By contrast, the case of this patient is slightly different because he is male, older than our patient, has no activator mutations on the KRAS gene, and has a single cerebellar metastasis. From a histological point of view, both tumors are adenocarcinomas, and both patients also have liver metastases.

All these data demonstrate the rarity of this case in the medical literature.

## Conclusions

Occult primary tumors are rare oncological pathologies encountered in daily oncological practice. This case report presents a rare case of an occult primary tumor with a suspicious primary tumor located in the left colonic flexure with synchronous metastases in the lung, liver, bone, and cerebellum in the context of determining the histopathological and immunohistochemical profile of this tumor from a pulmonary metastasis, although both flexible colonoscopy and PET-CT examinations showed a 15-mm lesion in the left colonic flexure. Furthermore, the histopathological examination of the colonic biopsy did not reveal any malignant proliferation.

A lot of improvements in the clinical outcomes of patients with colorectal cancer have been achieved in recent years, and this fact belongs to new systemic therapies, including immuno-oncology agents and targeted therapies. However, occult primary tumors and brain metastases from colorectal cancer are still rare cases seen in clinical oncology practice, making it imperative to find more targeted investigations that can facilitate the quickest diagnosis.
